# Iatrogenic Iron Overload in a Patient With Chronic Kidney Disease: Is There a Correlation Between Serum Ferritin and Liver Iron Concentration Determined by MRI T2*?

**DOI:** 10.7759/cureus.8914

**Published:** 2020-06-29

**Authors:** Mohammad Ali, Lina Okar, Phool Iqbal, Mohamed A Yassin

**Affiliations:** 1 Internal Medicine, Hamad Medical Corporation, Doha, QAT; 2 Family Medicine, Hamad Medical Corporation, Doha, QAT; 3 Internal Medicine, Hamad General Hospital, Doha, QAT; 4 Hematology and Oncology, Hamad General Hospital, Doha, QAT

**Keywords:** secondary iron overload, chronic kidney disease, liver iron concentration

## Abstract

Secondary iron overload in patients with chronic kidney disease (CKD) due to iatrogenic iron replacement is an emerging medical challenge. There are limited options to manage secondary iron overload in patients with CKD as most iron chelators are contraindicated due to low creatinine clearance. In addition to that, accuracy of serum ferritin in monitoring is questionable since it is affected by different variables including inflammation and liver disease. Moreover, correlation of serum ferritin with liver iron concentration (LIC) and heart iron concentration is not well studied in CKD patients. There is no established cut-off value in the current guidelines, and this warrants further investigation with more accurate methods.

There are few studies that evaluated the relationship between serum ferritin and LIC determined by different MRI protocols. Limited data in the literature concluded that a positive correlation exists between serum ferritin and LIC determined by MRI T2* and that serum ferritin more than 290 mcg/L is equivalent to severe iron overload on MRI T2*. However, we had a different observation of a patient with CKD on a prolonged course of iron replacement who was monitored with serum ferritin, and despite having a serum ferritin level of more than 1,000 mcg/L, LIC determined by MRI T2* was 5.3 mg/g of liver dry tissue, which is equivalent to mild iron overload.

This observation opens the door for further studies to examine the correlation between serum ferritin and LIC determined by MRI and to establish a safe cut-off value of serum ferritin so that further investigation would be indicated in patients with CKD.

## Introduction

It is a common practice to start patients on intravenous (IV) iron replacement without proper monitoring for iatrogenic iron overload. Iron overload among dialysis patients was widely considered to be more prevalent before the development of ESA, when blood transfusion was often used to treat anemia and IV iron was given without concomitant ESA. However, now it is increasingly recognized as a challenging clinical situation due to poor monitoring of iron status in these patients and the wide popularity of IV iron replacement. IV iron is more convenient in these patients as it can be infused during the dialysis sessions, has a superior efficacy over oral iron preparation in patients with concurrent inflammation, and helps to overcome functional iron deficiency commonly seen in chronic kidney disease (CKD) patients [1].

Monitoring for iron overload with serum ferritin and transferrin saturation (TSAT) represents the current clinical practice, although the accuracy of these markers is questionable as they are affected by different variables including inflammation and liver disease. Besides that, a cut-off value of serum ferritin at which iron overload should be suspected in CKD patients is not well defined. Moreover, the correlation of serum ferritin with liver iron concentration (LIC) and heart iron concentration is not well studied in CKD patients. Correlation between serum ferritin and LIC is well established in patients with transfusion-dependent anemia such as beta-thalassemia major [2]. Nevertheless, this correlation cannot be presumed to exist in CKD patients because secondary iron overload develops in a different mechanism.

The gold standard to assess for iron overload in the absence of liver cirrhosis is LIC determined by a liver biopsy. A standardized MRI protocol (FerriScan) is a reasonable non-invasive method to assess LIC with high sensitivity and specificity (94% and 100%, respectively). The normal LIC is between 0.4 and 2.2 mg/g of dry liver weight, LIC up to 7 mg/g of liver dry weight is not associated with significant adverse effect, and LIC > 15 mg/g is being consistently associated with liver cirrhosis. MRI T2* is another MRI protocol available for free, which provides the further advantage of monitoring for iron deposition in various vital organs, most importantly the liver and heart [3].

Iron deposition in body organs will lead to various complications including liver cirrhosis, arrhythmia, heart failure, diabetes mellitus, hypothyroidism, hypogonadism, and increase risk of infection [4-8]. Thus, comes the importance of more accurate screening methods such as MRI T2* to monitor for iatrogenic iron overload in CKD patients.

Here we present an interesting case of a patient with end-stage renal disease (ESRD) on hemodialysis. She was started on IV iron replacement and followed up with serum ferritin. She was referred to our clinic with serum ferritin > 1,000 mcg/L. MRI T2* was performed to evaluate for iron deposition in vital organs such as the liver and heart. Despite current data in the literature suggesting a correlation between LIC measured by MRI T2* and serum ferritin, we had a different observation as the LIC of this patient determine by MRI T2* was 5 mg/g of liver dry weight, suggestive of mild iron overload despite her high serum ferritin.

## Case presentation

A 54-year-old female known to have ESRD secondary to diabetic kidney disease and hypertension was started on hemodialysis three years ago. She was following up with nephrology clinic and was found to have anemia. Her Hb was 10.6 gm/dL her iron profile showed an iron level of 43 umol/L (normal range: 9.0-30.40), Total iron binding capacity (TIBC) of 35 umol/L (normal range: 45-80), TSAT of 23% (normal range: 15-45), transferrin of 1.41 gm/L (2.0-3.6), and ferritin of 567.9 mcg/L (normal range: 11-304). She was started on IV iron saccharate 100 mg three doses per week and ferrous sulfate 190 mg tablet daily for four years, and after that oral iron was stopped and she received IV iron supplementation with iron saccharate until she was referred to our clinic. She also received ESA monthly since she started the hemodialysis. She was following up in the nephrology clinic with monitoring of serum ferritin and TSAT. Over the past 16 months, her iron profile reflected a gradual increase in ferritin level from 10.7 to 1,618 and her TSAT increased from 14% to 94%.

**Figure 1:**
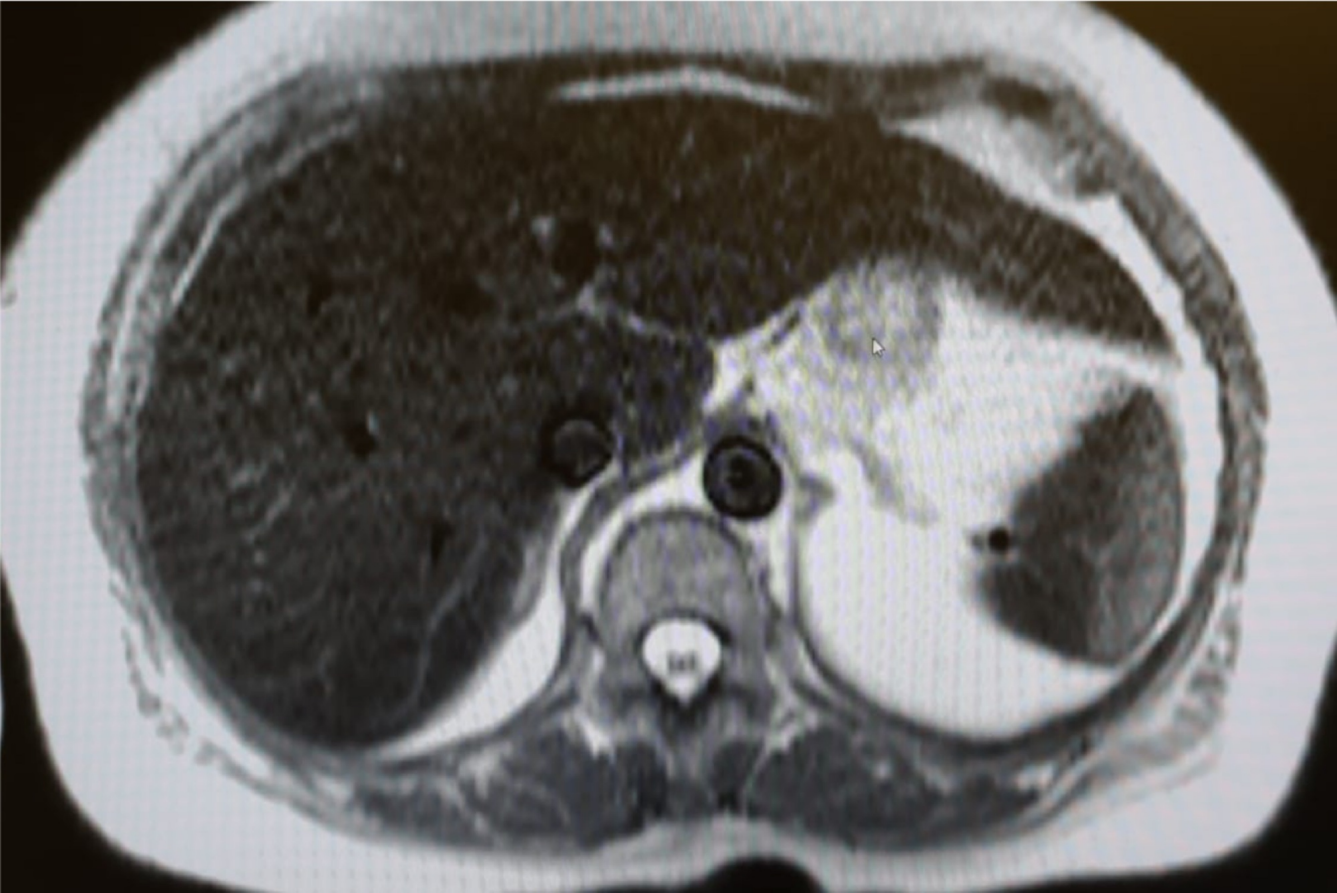
MRI T2* showing decreased liver signal intensity suggestive of iron overload; calculated LIC is 5.3 mg/g of dry liver weight, which proved mild iron overload. LIC, liver iron concentration

Suspected to have iatrogenic iron overload, she was referred to a hematology clinic. She denied any symptoms that would suggest a liver, heart, or endocrine gland dysfunction, and her physical examination was normal. To confirm the suspected diagnosis of secondary iron overload and to assess for iron deposition in vital body organs, MRI T2* was ordered, which showed iron deposition of <1.2 mg/g dry weight in the heart, which is considered normal, and LIC of 5.3 mg/g of liver dry weight, which is suggestive of mild iron overload (Figure 1).

To exclude iron deposition in vital endocrine glands, a full hormonal profile was ordered, which showed the following: thyroid-stimulating hormone (TSH) of 1.800 mIU/L (normal range: 0.38-5.33), free thyroxine (FT4) of 11.3 pmol/L (normal range: 8.4-19.1), and HbA1c of 7.5%.

The treatment decision was to hold iron replacement and to follow her closely while waiting for the consumption of excess iron by daily body needs. After stopping iron replacement, her serum ferritin went down to 588 mcg/L and TSAT decreased from 94% to 33% over one year.

## Discussion

Iron is stored in the body in the form of a ferritin complex, which is most commonly found in the liver, bone marrow, and spleen. However, the liver is the main storage reservoir, which is estimated to contain 70% of the total iron of the body. Since there is no physiological mechanism to eliminate excess iron when it accumulates in the body, any mechanism that increases the body uptake of iron beyond the daily needs will lead to iron overload. Iron overload might be due to primary or secondary causes. Primary iron overload is due to inherited disorders such as hereditary hemochromatosis. Secondary iron overload can occur in hematological diseases and in CKD patients. In the context of hematological diseases, iron accumulates due to different mechanisms including chronic transfusion, increased gastrointestinal absorption, chronic hemolysis, and underlying genetic defects, leading to an increase in gastrointestinal absorption of iron [3]. In the context of CKD, secondary iron overload develops due to iatrogenic iron replacement. Multiple factors play a role in the development of iatrogenic iron overload in ESRD patients. This includes the current clinical practice based on the 2012 KDIGO (Kidney Disease: Improving Global Outcomes) guidelines recommendations, which encouraged the use of IV iron in hemodialysis patients. Besides that, the poor follow-up and monitoring of iron storage in those patients is an important contributing reason that must be considered [9].

Iatrogenic iron overload is an emerging complication in CKD patients, and the current guidelines advocate for certain measures to prevent this complication. The 2012 KDIGO guidelines recommend testing for iron status (TSAT and ferritin) more frequently when monitoring response after a course of IV iron. Both the American and Australian guidelines recommend caution with the routine administration of IV ferritin if the serum ferritin is >500 mcg/L, yet the upper limit of a safe serum ferritin level remains unresolved [10]. Despite these measures, the guidelines do not answer important questions about the accuracy of serum ferritin in CKD patients, especially those with concomitant inflammation or infection, the cut-off value at which further investigation with MRI is recommended, and the correlation between serum ferritin and LIC determined by MRI.

In CKD patients, the cut-off value of serum ferritin/TSAT at which further investigation with MRI is recommended is not well established [10]. The gold standard to assess for iron overload in the absence of liver cirrhosis is LIC determined by liver biopsy. A standardized MRI protocol (FerriScan) is a reasonable non-invasive method to assess LIC with high sensitivity and specificity (94% and 100%, respectively). The normal LIC is between 0.4 and 2.2 mg/g of dry liver weight, LIC up to 7 mg/g of liver dry weight is not associated with significant adverse effect, and LIC > 15 mg/g is being consistently associated with liver cirrhosis. MRI T2* is another MRI protocol available for free, which provides the further advantage of monitoring for iron deposition in various vital organs, most importantly the liver and heart [3].

The correlation between serum ferritin and LIC is well established in transfusion-dependent thalassemia patients [2]. However, this correlation cannot be assumed to apply to CKD patients due to the different mechanism of iron deposition in the body and the various variables that can affect serum ferritin in CKD patients, such as inflammation. Establishing this correlation in CKD patients, if it exists, is of utmost importance as it can decrease the need for further investigation with more expensive methods such as MRI. There are a few studies that evaluated the relationship between serum ferritin and LIC using different MRI protocols including SQUID, FerriScan, and MRI T2*. Canavese et al. used SQUID, which is an expensive MRI machine available in the research center, to evaluate for this correlation and found that serum ferritin is roughly representative of the liver iron store at least in hemodialysis patients without overt inflammation. The study concluded that when serum ferritin value exceeds 500 mcg/L, the risk of having moderate-to-severe storage iron overload is increased 10 times [11]. Another study by Ferrari et al. evaluated the correlation between different iron markers and LIC determined by FerriScan and showed no correlation between serum iron markers including serum ferritin/TSAT and LIC [10]. Both of these studies are limited by the small sample size which limits the generalizability of their result. Beside that, these studies used different MRI methods than MRI T2*, as used in our patient. The studies by Ghoti et al. and Rostoker et al. used MRI T2* to evaluate if a correlation exists between serum ferritin and LIC. Both studies concluded that a positive correlation exists between serum ferritin and LIC determined by MRI T2*, and suggested a serum ferritin cut-off value of 290 mcg/L, which warrants further investigation with MRI [12,13]. We had a different observation as our patient had serum ferritin more than 1,000 mcg/L, whereas LIC determined by MRI T2* was 5.3 mg/g of liver dry tissue, which is equivalent to mild iron overload. This observation questions the conclusion of previous studies about the existence of a positive correlation between serum ferritin and LIC, and opens the door for further studies with a large sample size to examine this correlation.

The treatment of secondary iron overload in CKD patients is challenging as many iron chelators are contraindicated due to low creatinine clearance. Stopping iron replacement and close observation of the patient is the only option in many cases. Besides that, secondary iron overload can lead to various complications in these patients, including increase risk of infection, and some studies suggest that an increase in serum ferritin is a marker of increased risk of cardiovascular disease. Moreover, iron deposition in body organs will lead to liver cirrhosis and endocrine gland dysfunction, leading to diabetes mellitus, hypogonadism, and hypothyroidism [3].

## Conclusions

The limitation of treatment options and the various complications that might result from secondary iron overload in CKD patients highlight the importance of prescribing iron replacement wisely to these patients and monitoring them closely. Serum ferritin and TSAT are suggested by current guidelines to monitor for secondary iron overload in CKD patients. There are limited data published on the correlation between serum ferritin and LIC measured by different MRI protocols. The current data in the literature suggest a positive correlation between serum ferritin and LIC determine by MRI T2* and that a ferritin value of more than 290 mcg/L is equivalent to severe iron overload. However, we had a different observation that questions the existence of this correlation, as our patient had a serum ferritin level of >1,000 mcg/L and his LIC measured by MRI T2* was suggestive of mild iron overload. We believe that more studies are needed to examine the correlation between serum ferritin and LIC in CKD patients. This will help to determine if a positive correlation exists and at which cut-off value further investigation with MRI will be recommended.
